# Challenge in the diagnosis of pulmonary strongyloidiasis

**DOI:** 10.31744/einstein_Journal/2019AI4441

**Published:** 2018-12-17

**Authors:** Andrea dos Santos Pereira, Alexandre Gimenes Marques, André Mario Doi, Marinês Dalla Valle Martino, Paula Célia Mariko Koga, Vivian Renata Chiarato, Liang Fung, Rima Batah Martins, Jacyr Pasternak

**Affiliations:** 1 Hospital Israelita Albert Einstein, São Paulo, SP, Brasil

A 33-years-old man was admitted to the intensive care unit because of alcoholic cirrhosis, cocaine toxicity, and septic shock to be accurately assessed. Further tests were requested.

His X-ray did not show compatible patterns with pulmonary strongyloidiasis, blood culture was positive for enterobacteria that produce Klebsiella pneumoniae carbapenemase (KPC), and discrete eosinophylin hemogram and tracheal secretion culture were observed.

In culture requested 6 days after interventions, a characterized and uncommon pattern polymicrobial flora growth was observed in culture medium. The phenomeum “traits” was observed (Figures 1 and 2), which suggests that something was moving between agar colonies, as reported by other authors.^(^ ^1^ ^)^ In this culture, a fresh research was conducted and results confirmed the presence of larvae of *Strongyloides stercoralis* (Figure 3).

**Figure 1:**
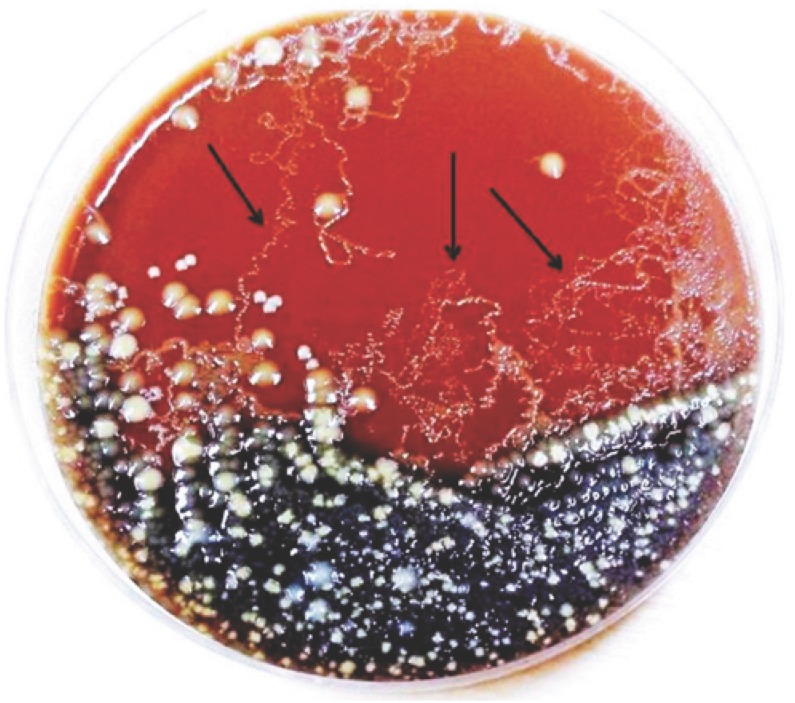
Blood agar plate after a 24-hour incubation Line drawing among bacterial colonies (arrows).

**Figure 2:**
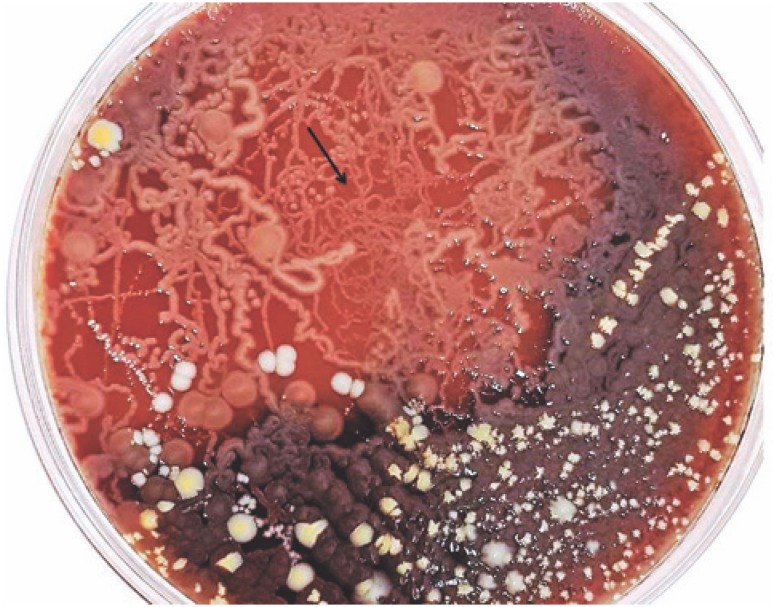
Blood agar plate after a 48-hour incubation Line drawing among bacterial colonies (arrows).

**Figure 3:**
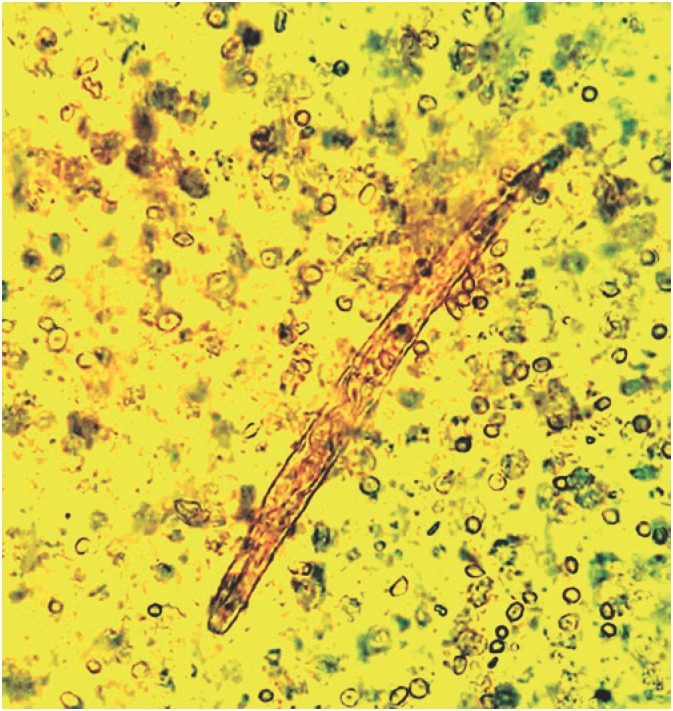
*Strongyloides stercoralis* larvae in fresh tracheal secretion.

After this result, both the department of nosocomial infection and medical team were promptly communicated. A treatment with ivermectine was administered. Although specific therapy for disseminated Strongyloidiasis was started, the patient died due to advanced clinical complications.

Strongyloidiasis is an infection caused by *Strongyloides stercoralis* helminthic parasite, which distribution occurs mainly in tropical and subtropical countries in rural and low-socioeconomic status areas. The infection has two life cycles: (1) free life cycle in which the larvae can penetrate the intact skin after contact with contaminated soil, and (2) in which the larvae migrates to the lungs, after, to pharynx, which is them swallowed, and become adult in the intestine and is released in the stools.

Both the physician and the microbiology laboratory should carefully follow-up the microorganism growth in immunosuppressed patients, which is uncommon in culture of immunocompetent patients. In our case, the microscopy of fresh material was fundamental to elucidate the diagnosis, once the *strongyloides* research was not initially requested. This infection can be fatal for immunosuppressed individuals.^(^ ^2^ ^,^ ^3^ ^)^

## References

[1] Kia EB, Mahmoudi M, Zahabiun F, Meamar AR (2007). An evaluation on the efficacy of agar plate culture for detection of strongyloides stercoralis. Iranian J Parasitol..

[2] Centers for Disease Control and Prevention (CDC) (2017). DPDx - Laboratory identification of parasites of public health concern. Strongyloidiasis [Internet].

[3] Ash LR, Orihel TC (1997). Atlas of Human Parasitology.

